# Investigation of femtosecond laser induced ripple formation on copper for varying incident angle

**DOI:** 10.1063/1.5020029

**Published:** 2018-01-10

**Authors:** Craig A. Zuhlke, George D. Tsibidis, Troy Anderson, Emmanuel Stratakis, George Gogos, Dennis R. Alexander

**Affiliations:** 1 Department of Electrical and Computer Engineering, University of Nebraska-Lincoln, Lincoln, Nebraska 68588, USA; 2 Institute of Electronic Structure and Laser (IESL), Foundation for Research and Technology (FORTH), N. Plastira 100, Vassilika Vouton, 70013 Heraklion, Crete, Greece; 3 Materials Science and Technology Department, University of Crete, 71003 Heraklion, Greece; 4 Department of Mechanical and Materials Engineering, University of Nebraska-Lincoln, Lincoln, Nebraska 68588, USA

## Abstract

The hydrodynamic mechanisms associated with the formation of femtosecond laser induced ripples on copper for two angles of incidence are reported. Laser pulse length used for this work is 35 fs. A revised two-temperature model is presented that comprises transient changes of optical characteristics during the irradiation with femtosecond pulses to model relaxation processes and thermal response in bulk copper. The theoretical model takes into account the fluid flow dynamics that result in ripple periods shorter than the wavelength of the surface plasmon polaritons. Theoretical and experimental results are reported for incident angles of 0**°** and 45**°** relative to the surface normal. There is agreement between the experimentally measured and the theoretically predicted ripple periodicity for 50 pulses at 0**°** incidence. By contrast, for 100 pulses at 0**°** incidence, and 50 and 100 pulses at 45**°** incidence, the experimentally measured ripples have a larger period than the one predicted by the model while the trends in period with increased incident angle, and increased fluence are in agreement between the experimental and the theoretical results.

The use of femtosecond lasers to produce self-organized micro and nanoscale surface structures on metals is a rapidly developing technology with a range of applications including enhancing heat transfer,^1–3^ producing antibacterial surfaces,^4^ and altering the wetting^2,5^ and optical absorption^6,7^ properties of materials. There has been a large amount of theoretical and experimental work aimed at understanding formation mechanisms^8–14^ and using this understanding to tailor surfaces for specific applications. Laser induced ripples are a class of surface structures that are often the focus of theoretical modeling studies because, in general, they develop with fewer pulses interacting with the surface and are less complex than other structures formed with additional pulse counts and/or at higher fluence values. A quantitative interpretation of the dynamics leading to self-organized surface structure formation from pulsed laser interaction with materials can enable controllability in the production of structures with well-defined properties (optical/morphological, etc.). Although most studies emphasize the role of surface plasmon (SP) excitation or scattering from a rough surface (the most prominent scenario), there are still unexplored mechanisms related to the (i) optical property changes during the pulse duration, (ii) hydrodynamics, and (iii) how the surface plasmon polariton (SPP) and the resulting ripple (upon resolidification) are affected when the angle of incidence is not normal to the surface. In this work, we present a theoretical model, along with experimental results on the ripple periodicity on polished copper (Cu) for pulses incident at 0**°** and 45**°** relative to the surface normal.

Cu 110 samples with a thickness of 812.8 μm and an initial mean surface roughness of 29 nm are used in the experimental work. The laser used for this work was a Coherent, Astrella femtosecond laser which produces 35 fs pulses at a 1 kHz repetition rate with a maximum pulse energy of 6 mJ. A phase locked chopping wheel in combination with a fast laser shutter (Uniblitz, VS35) was used to control sample illumination down to single pulses. The pulse to pulse stability of the laser is ± 0.49%, and was measured using a pyroelectric detector (818E-10–25-F) and a power meter capable of capturing and calculating statistical data (1936-R). The position of the sample surface relative to the focal volume was controlled using Melles Griot Nanomotion II stages with three axis of motion. The energy of the pulses was controlled using a half waveplate in combination with a polarizer. All experimental work was completed in air. The incident angle of the laser pulses was controlled by keeping the laser optics stationary and tilting the translation stage with the Cu sample attached. The incident angle of the pulses was defined relative to the surface normal. Cu samples were imaged in a Quanta 200 FEG Environmental Scanning Electron Microscope (SEM) with a maximum resolution of 3 nm. The spot size of the pulses at the sample surface was determined using the method outlined by Liu.^15^ The diameter of the ablation craters as function of pulse energy for 100 incident pulses was measured in the SEM. The edge of the ablation crater was considered to be at the transition between ripples and the polished surface. The stated spot size values are the average from 5 sets of craters. For the ablation craters produced at normal incidence, the spot radius of the pulses was 22 μm. For 45 degree incidence angle the ablation craters were elliptical and the spot radius of the minor axis was 20 μm.

The period of the ripples for various fluence, pulse count and angle of incidence values was determined by taking the Fast Fourier Transform (FFT) of SEM images using MATLAB. The FFT was applied to a square SEM image of the center portion of the ablation crater. For 45 degree illuminations the FFT region included about 7 ripples and for the normal incidence about 16 ripples were included. SEM images of the ablation craters were taken at 20,000 × magnification with a resolution of 4096 × 3773 pixels. The pixel resolution of the images was 3.6 nm/pixel. SEM images were padded with 2^14^ zeros prior to taking the FFT, which resulted in an FFT resolution of 2.3 nm per data point. The period of the ripples was taken to be the peak value of the FFT along the axis oriented perpendicular to the ripples. Representative SEM images for both normal incidence and 45**°** incidence are included in Fig. 1.

As noted in previous works, ripple formation on solids is due to combined electrodynamical^16–18^ and hydrodynamical effects.^14,19–21^ With respect to the role of electrodynamics, SP excitation is induced on metals as *Re(ε)* < −1, where *ε* stands for the dielectric constant of the irradiated material. The SP excitation is, mainly, manifested by the production of surface waves of variable periodicity and polarization that depend on the material^14,19, 20,22–30^ and the incident angle^31,32^ *θ*. The predicted SP wavelength, *Λ*_*S*_, is provided by Λ_*S*_ = *λ*_*L*_/(*η* – sin*θ*),^33^ where $\eta =\text{Re}\sqrt{\varepsilon \varepsilon_{d}/\left(\varepsilon +\varepsilon_{d}\right)}$,^31^ *εd* is the dielectric constant of the ambient (for air, *ε*_*d*_ *=* 1) and *λ*_*L*_ stands for the laser beam wavelength. Although the SP wave periodicity is mainly determined by *Λ*_*S*_, the experimentally observed frequency of the periodic structures is slightly different than *Λ*_*S*_, which indicates that material phase transition is required to describe the discrepancy.^34^ An absence of periodic structure formation for irradiation with one pulse (number of pulses (*NP*) = 1) is anticipated since an initially flat surface leads to a free-space photon having less momentum than an SP. Hence, a surface plasmon polariton (SPP) cannot be excited on a flat surface since the laser wavelength dispersion curve does not intersect the surface plasmon dispersion curve.^35^ On the other hand, even a small corrugation deviation on the surface or the presence of periodical structures facilitates the SPP excitation.^18,35^ Tsibidis^19^ reports that although the first pulse is not sufficient to produce ripples, the laser beam conditions induce a phase transition that leads to the formation of a crater. This initial instability serves as the precursor for SP excitation in subsequent laser pulse-matter interactions. Therefore, for a complete investigation of the process of ultrashort pulse surface modification, it is necessary to explore the variation of the dielectric constant of the material (energy absorption), electron excitation and relaxation processes, and the phase transition followed by a resolidification process.

With respect to the change of *ε* for repetitive irradiation (i.e. increasing *NP*), correlation of morphological characteristics of the irradiated zone (corrugation amplitude, *δ* and ripple periodicity, *Λ*) with the magnitude of the longitudinal wavevector of the SPP requires a systematic analysis of the propagation of the respective electromagnetic field.^20,35^ The solution of the Maxwell’s equations, along with the requirement of continuity of the tangential component of the electric field $\vec{E}$ and normal component of $\varepsilon \vec{E}$ on the boundary defined by the surface profile, allows determination of the spatial distribution of the electric field and derivation of the dispersion relations. To estimate the optimal laser-grating coupling, the combination of maximum amplitude *δ* and resonant length *Λ*_*S*_ are computed that yield enhanced longitudinal electric field inside the irradiation zone.^20^ Previous studies on SP wavelength dependence on the NP indicate that successive irradiation leads to a decrease of the periodicity of the ripples with increasing *NP*.^20,22,36,37^

It has been shown that for laser irradiation of metals, such as Au or Cu, with very short pulses, optical properties vary substantially during the pulse duration, which is expected to influence the energy absorption of the irradiated material.^34,38,39^ The computation of the absorption coefficient *α* and reflectivity *R* is performed through the computation of the dielectric constant by assuming the extended Lorentz-Drude model with four Lorentzian terms based on the analysis of Rakic *et* al.^40^

(1) $$\varepsilon \left(\omega_{L}\right)=1-\frac{f_{0}\omega_{p}^{2}}{\omega_{L}^{2}-i\gamma \omega_{L}}+\sum_{j=1}^{k=4}\frac{f_{j}\omega_{p}^{2}}{\omega_{j}^{2}-\omega_{L}^{2}+\text{i}\omega_{L}\Gamma_{j}}$$

where *ω*_*L*_ is the laser frequency (*ω*_*L*_ = 2.3562 × 10^15^ rad/s for 800 nm, which corresponds to photon energy equal to 1.55 eV), $\sqrt{f_{0}\omega_{p}}$ is the plasma frequency associated with an oscillator strength *f*_*0*_ and damping constant *γ* (equal to the reciprocal of electron relaxation time), *f*_*0*_ = 0.575, *ħω*_*p*_ **= 9.01 eV**, *ω*_*1*_ = 0.291 eV, *ω*_*2*_ = 42.957, *ω*_*3*_ = 5.3, *ω*_*4*_ = 11.18, *Γ*_*1*_ = 0.378 eV, *Γ*_*2*_ = 1.056, *Γ*_*3*_ = 3.213, *Γ*_*4*_ = 4.305, *f*_*1*_ = 0.061, *f*_*2*_ = 0.104, *f*_*3*_ = 0.723, and *f*_*4*_ = 0.638. The temporal dependence of the dielectric constant and the optical parameters come from the electron relaxation time, which is the sum of the electron-electron and electron-phonon collision rates, *A(T*_*e*_*)*^*2*^ and *BT*_*L*_, respectively (*A =* 1.28×10^7^ s^−1^K^−2^, *B* = 1.23×10^11^ s^−1^K^−1^ for Cu^41^), where *T*_*e*_ and T_*L*_ are the electron and lattice temperatures respectively. Hence, the dynamic character of the optical properties is incorporated in the simulations through the temporal change of *γ*. On the other hand, the spatio-temporal distribution of *T*_*e*_ and *T*_*L*_ are derived by the use of the traditional two-temperature model:^42^

(2a) $$C_{e}\frac{\partial T_{e}}{\partial t}=\vec{\nabla }\cdot \left(k_{e}\vec{\nabla }T_{e}\right)-G_{eL}\left(T_{e}-T_{L}\right)+S\left(x,\;y,\;z,\;t\right)$$

(2b) $$C_{L}\frac{\partial T_{L}}{\partial t}=G_{eL}\left(T_{e}-T_{L}\right)$$

Where $S\left(x,\;y,\;z,\;t\right)=\frac{\sqrt{4log2}}{\sqrt{\pi }\tau_{p}}\alpha \left(x,\;y,\;z,\;t\right)\left(1-R\left(x,\;y,\;z=0,\;t\right)\right)E_{p}\text{exp}\left(-4log2{\left(\frac{t-3\tau_{p}}{\tau_{p}}\right)}^{2}\right)\int_{0}^{z}\text{exp}\left(-\alpha \left(x,\;y,\;z^{\prime },\;t\right)z^{\prime }\right)dz^{\prime }$, the subscripts *e* and *L* are associated with electrons and lattice, respectively, *k*_*e*_ is the thermal conductivity of the electrons, *C*_*e*_ and *C*_*L*_ are the heat capacity of electrons and lattice, respectively, *G*_*eL*_ is the electron-phonon coupling constant, and *S* stands for the source term due to laser heating. It is the irradiation of a periodically modulated surface that leads to an inhomogeneous distribution of the laser beam energy. Hence, the periodic character of the varied deposition of energy is also transferred to all parameters in the model including the temperature. As a result, when material melts, spatially periodic fluid phenomena will be developed leading to resolidification in periodic fringes.^19^ On the other hand, to introduce phase transitions that cause the bulk temperature to exceed the melting temperature (*T*_*m*_ = 1357 K), Eq. 2b is modified to include a phase change in the solid-liquid interface:

(3) $$\left(C_{L}^{\left(m\right)}\pm L_{m}\delta \left(T_{L}-T_{m}\right)\right)\frac{\partial T_{L}}{\partial t}=\vec{\nabla }\cdot \left(K_{L}^{\left(m\right)}\vec{\nabla }T_{L}\right)+G_{eL}\left(T_{e}-T_{L}\right)$$

where *L*_*m*_ is the latent heat of fusion (~205 kJ/kg for Cu). $C_{L}^{\left(m\right)}$ and $K_{L}^{\left(m\right)}$ stand for the heat capacity and thermal conductivity of the liquid phase, respectively.^43^ Due to large electron temperatures (~12×10^4^ K) reached for the laser beam conditions of the simulation (pulse duration τ_*p*_ = 35 fs, laser wavelength *λ*_*L*_ = 800 nm, fluence *E*_*p*_ = 0.30 J/cm^2^, irradiation spot radius *R*_*0*_ = 20 μm) and the variation of the energy absorption within the irradiation time as a result of the temporal variation of the optical properties, the thermophysical properties of the material will be very sensitive to electron temperatures. Hence, to provide an accurate description of the underlying mechanism after irradiation with ultrashort pulses, the thermophysical properties that appear in Eqs. (2) and (3) will be temperature dependent, based on Lin *et al*.^44^ The fluid transport is described through the treatment of the molten material as an incompressible Newtonian fluid and the use of the conventional Navier-Stokes equation:

(4) $$\rho_{L}^{\left(m\right)}\left(\frac{\partial \vec{u}}{\partial t}+\vec{u}\cdot \vec{\nabla }\vec{u}\right)=\vec{\nabla }\cdot \left(-P1+\mu \left(\vec{\nabla }\vec{u}\right)+\mu {\left(\vec{\nabla }\vec{u}\right)}^{T}\right)$$

Where $\vec{u}$ is the velocity of the fluid, $\rho_{L}^{\left(m\right)}$ is the liquid density, *μ* is the liquid viscosity,^45^ and *P* is the liquid pressure.

Simulation results on a staggered grid (see a more analytical description in Tsibidis *et* al.^19^) are presented in Fig. 2. Resolidification of the capillary material flow leads to the production of the rippled profile shown in Fig. 2a for *NP* = 50. The results illustrated in Fig. 2a show the formation of periodical structures with periodicity *Λ* = 1604 nm at incident angle *θ* = 45^0^. Similarly, for *NP* = 100, the formation of ripples is illustrated in Fig. 2b. It is evident that for a larger *NP*, the crater depth is larger while the periodicity decreases (*Λ* = 1559 nm). This monotonicity is predicted from the theoretical model based on the correlation of the depth of the corrugated region and the periodicity of the SP. Furthermore, it is evident from Fig. 2 that irradiation at a non-zero angle induces an elliptical spot. Although not shown here, the experimentally produced ablation craters for a 45° incident angle were also elliptical.

In order to compare the theoretical and experimental results for various values of the fluence, pulse count and incident angle, the periodicity of ripples at the center of the ablation crater was recorded for the theoretical results for 50 and 100 pulses as a function of fluence from 0.25 J/cm^2^ to 0.45 J/cm^2^ for both 0° and 45° incidence. The experimentally measured periodicity was also recorded for 50 and 100 pulses for both 0° and 45° incidence over the fluence range for which ripples were visible in the center of the craters. Both the theoretical and the experimental results are included in Fig. 3a and Fig. 3b. Values of the experimental data points in Fig. 3 correspond to the mean ripple period for 5 – 10 ablation craters. The error bars indicate the standard deviation for the ripple period. Ten ablation craters were produced for each data point, however, due to localized surface defects some ablation craters did not have well defined ripples. The ablation craters were analyzed on a case by case basis and data from craters that did not have well defined ripples were not included.

It is evident that there is agreement between the experimentally measured and the theoretically predicted ripple period for 50 pulses incident normal to the surface. Regarding the other pulse count and incident angle combinations reported in this work, the theoretical model predicts similar trends to those resulting from experimental measurements. An increase in ripple periodicity for pulses incident at 45° versus 0° is noted by comparing results in Fig. 3(a) with Fig. 3(b). Data illustrated in Fig. 3 indicate that there is a weak increase in ripple periodicity with increased fluence that is predicted theoretically and confirmed experimentally. Similar trends were previously predicted using a simpler model.^31^ The data plotted in Fig. 3 correspond to the range in fluence for which ripples are experimentally observed, however, the theoretical model predicts ripples at fluence values larger than the experimentally observed upper limit. There are several differences between the theoretical model and the experimental conditions that could account for discrepancies in the results. First, the theoretical model assumes that consecutive pulses always irradiate a pure Cu surface in vacuum, however, laser processing is performed in air which suggests that the surface oxidizes after irradiation. An oxidized surface (i.e. CuO) is characterized by a very large dielectric constant^46^ that is expected to reduce the simulated ripple periodicity considering the aforementioned expression relating *ε* with the SPP wavelength. Second, the theoretical model does not consider possible formation of surface roughness on ripples, which suggests a need for introducing an effective refractive index that will lead to a reduced computed ripple periodicity.^31^ Third, the theoretical model assumes an, initially, smooth surface, while experimentally the surface has some initial roughness. Although, the incorporation of the aforementioned reasons would rather indicate that the theoretical period values should be larger than the experimental values, a more thorough systematic investigation is required to quantify the influence of the experimental conditions on the periodicity of the ripples. It should also be noted that experimental estimation of the threshold fluence value for ripple formation is observed to be lower for 45° incidence than normal incidence. This is not predicted by the theoretical model. However, for 45° incidence pulses, the edge of the ablation craters are not sufficiently defined compared to those produced using pulses incident normal to the surface. The increased error associated with the spot size measurements for 45° incidence versus 0° incidence could account for the shift in threshold to lower fluence values.

In conclusion, a revised two-temperature model is presented that comprises transient changes of optical material characteristics during the irradiation with femtosecond pulses to describe thermal response of polished bulk Cu samples undergoing phase transitions. The theoretical model takes into account the fluid dynamics that yield subwavelength ripples. Theoretical and experimental results exhibit a similar trend for the periodicity for incident angles of 0° and 45° with increasing fluence. There is also agreement between the experimentally measured and the theoretically predicted ripple period for 50 pulses incident normal to the surface. Discrepancies in the values of the periodicity of the ripples between the theoretical model and experimental observations are attributed to various assumptions considered in the theoretical model that need to be explored further.

## Figures and Tables

**FIG. 1.**
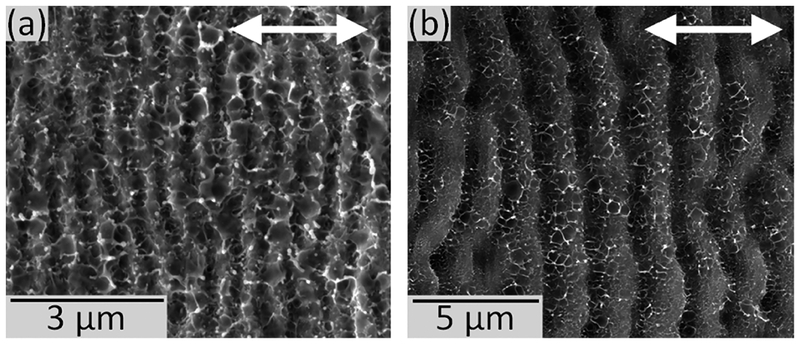
SEM images of ripples at (a) normal incidence formed using a peak fluence of 0.26 J/cm^2^ and (b) 45° incidence developed using a peak fluence of 0.25 J/cm^2^. The double-ended arrows indicate the polarization of the incident ablation pulses.

**FIG. 2.**
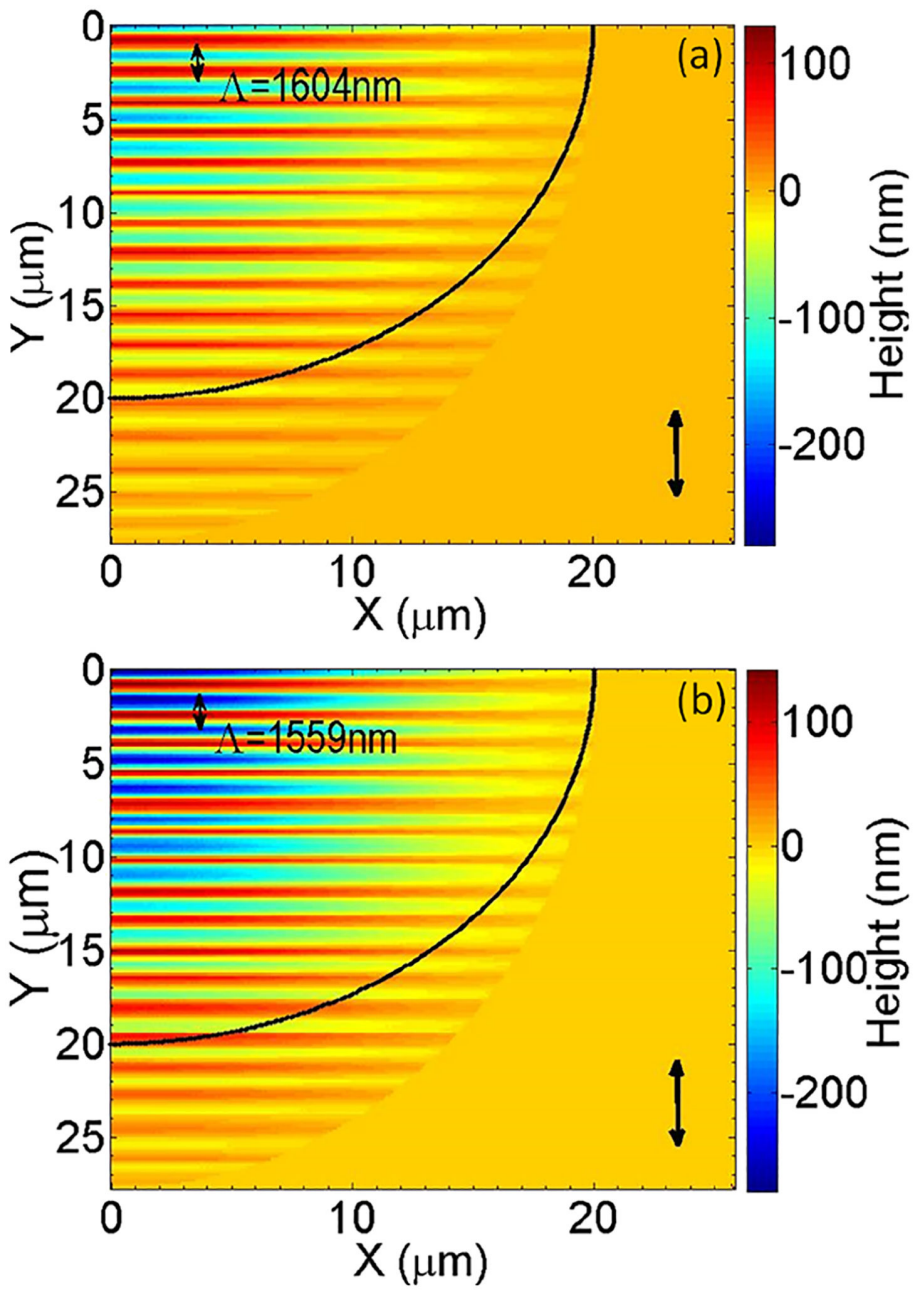
(a) Surface profile (quadrant) for *NP* = 50, and (b) surface profile (quadrant) for *NP* = 100, (*E*_*p*_ = 0.30 J/cm^2^, *τ*_*p*_ = 35 fs, *θ* = 45^0^). The *black vertical* double arrow indicates the polarization of the laser beam while the *black* line shows the size of the irradiation beam.

**FIG. 3.**
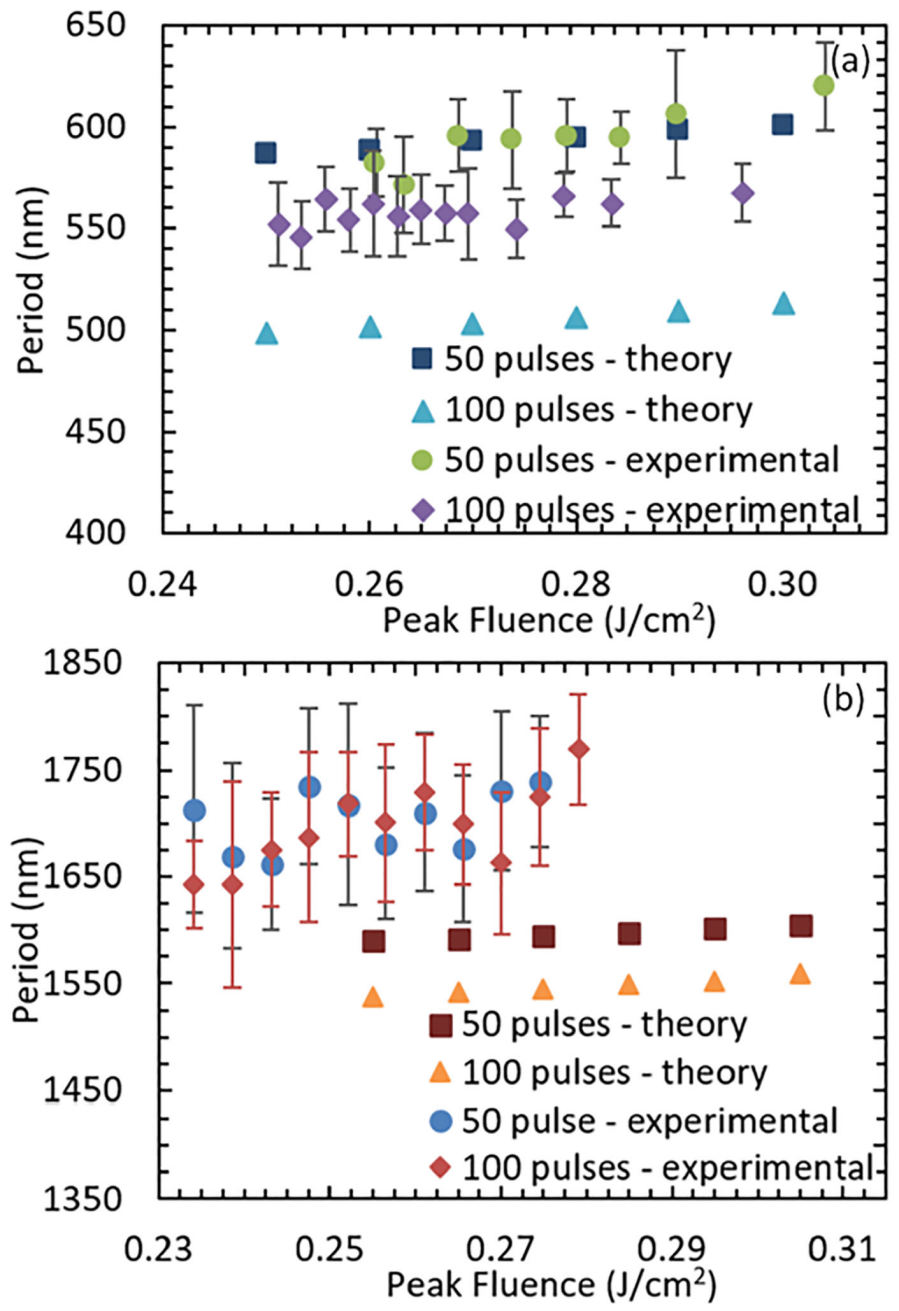
Theoretical and experimental results for the period of ripples on Cu for pulses incident at (a) 0° and (b) 45° relative to the surface normal over a range of fluence values.

## References

[1] Kruse C, Tsubaki A, Zuhlke C, Anderson T, Alexander D, Gogos G, and Ndao S, Appl. Phys. Lett 108, 51602 (2016). 10.1063/1.4941081 PMC6288672 30546153

[2] Kruse CM, Anderson T, Wilson C, Zuhlke C, Alexander D, Gogos G, and Ndao S, Int. J. Heat Mass Transf 82, 109 (2015). 10.1016/j.ijheatmasstransfer.2014.11.023 PMC6235449 30449897

[3] Kruse C, Anderson T, Wilson C, Zuhlke C, Alexander D, Gogos G, and Ndao S, Langmuir (2013). 10.1021/la401936w 23799305

[4] Fadeeva E, Truong VK, Stiesch M, Chichkov BN, Crawford RJ, Wang J, and Ivanova EP, Langmuir 3012 (2011). 10.1021/la104607g 21288031

[5] Zuhlke CA, Anderson TP, Li P, Lucis MJ, Roth N, Shield JE, Terry B, and Alexander DR, Proc. SPIE 93510J (2015).

[6] Singh N, Alexander DR, Schiffern J, and Doerr D, J. Laser Appl 18, 242 (2006).

[7] Vorobyev AY and Guo C, Appl. Phys. Lett 92, 41914 (2008).

[8] Peng E, Tsubaki A, Zuhlke CA, Wang M, Bell R, Lucis MJ, Anderson TP, Alexander DR, Gogos G, and Shield JE, Appl. Phys. Lett 108, 31602 (2016). 10.1063/1.4939983 PMC6225069 30416199

[9] Peng E, Tsubaki A, Zuhlke CA, Wang M, Bell R, Lucis MJ, Anderson TP, Alexander DR, Gogos G, and Shield JE, Appl. Surf. Sci 396, 1170 (2017). 10.1016/j.apsusc.2016.11.107 PMC6218947 30410203

[10] Zuhlke CA, Alexander DR, Bruce JC, Ianno NJ, Kamler CA, and Yang W, Opt. Express 18, 4329 (2010). 20389444 10.1364/OE.18.004329

[11] Zuhlke CA, Anderson TP, and Alexander DR, Opt. Express 21, 8460 (2013). 23571936 10.1364/OE.21.008460

[12] Zuhlke CA, Anderson TP, and Alexander DR, Appl. Phys. Lett 103, 121603 (2013).

[13] Zuhlke CA, Anderson TP, and Alexander DR, Appl. Surf. Sci 21, 8460 (2013). 10.1016/j.apsusc.2016.11.107 PMC6218947 30410203

[14] Tsibidis GD, Fotakis C, and Stratakis E, Phys. Rev. B 92, 41405 (2015).

[15] Liu JM, Opt. Lett 7, 196 (1982). 19710869 10.1364/ol.7.000196

[16] Bonse J, Munz M, and Sturm H, J. Appl. Phys 97, 13538 (2005).

[17] Huang R and Suo Z, J. Appl. Phys 91, 1135 (2002).

[18] Sipe JE, Young JF, Preston JS, and van Driel HM, Phys. Rev. B 27, 1141 (1983).

[19] Tsibidis GD, Barberoglou M, Loukakos PA, Stratakis E, and Fotakis C, Phys. Rev. B 86, 115316 (2012).

[20] Tsibidis GD, Skoulas E, and Stratakis E, Opt. Lett 40, 5172 (2015). 26565827 10.1364/OL.40.005172

[21] Varlamova O, Costache F, Ratzke M, and Reif J, Appl. Surf. Sci 253, 7932 (2007).

[22] Bonse J and Krüger J, J. Appl. Phys 108, 34903 (2010).

[23] Höhm S, Rosenfeld A, Krüger J, and Bonse J, J. Appl. Phys 112, 14901 (2012).

[24] Derrien TJ-Y, Itina TE, Torres R, Sarnet T, and Sentis M, J. Appl. Phys 114, 83104 (2013).

[25] Barberoglou M, Tsibidis GD, Gray D, Magoulakis E, Fotakis C, Stratakis E, and Loukakos PA, Appl. Phys. A 113, 273 (2013). 10.1364/OE.21.018501 23938722

[26] Tsibidis GD, Stratakis E, and Aifantis KE, J. Appl. Phys 111, 53502 (2012).

[27] Tsibidis GD, Stratakis E, Loukakos PA, and Fotakis C, Appl. Phys. A Mater. Sci. Process 114, 57 (2014).

[28] Colombier JP, Garrelie F, Faure N, Reynaud S, Bounhalli M, Audouard E, Stoian R, and Pigeon F, J. Appl. Phys 111, 24902 (2012).

[29] Garrelie F, Colombier JP, Pigeon F, Tonchev S, Faure N, Bounhalli M, Reynaud S, and Parriaux O, Opt. Express 19, 9035 (2011). 21643157 10.1364/OE.19.009035

[30] Wang J and Guo C, Appl. Phys. Lett 87, 251914 (2005).

[31] Hwang TY and Guo C, J. Appl. Phys 108, 73523 (2010).

[32] Hwang TY and Guo C, J. Appl. Phys 110, 73521 (2011).

[33] Bonch-Bruevich AM, Opt. Eng 31, 718 (1992).

[34] Tsibidis GD, Appl. Phys. Lett 104, 51603 (2014).

[35] Raether H, Surface Plasmons on Smooth and Rough Surfaces and on Gratings (Springer-Verlag, Berlin, New York, 1988).

[36] Rudenko A, Colombier J-P, and Itina TE, Phys. Rev. B 93, 75427 (2016).

[37] Bonse J, Höhm S, Rosenfeld A, and Krüger J, Appl. Phys. A 110, 547 (2012).

[38] Ren Y, Chen JK, and Zhang Y, J. Appl. Phys 110, 113102 (2011).

[39] Ren Y, Chen JK, Zhang Y, and Huang J, Appl. Phys. Lett 98, 191105 (2011).

[40] Rakić AD, Djurišić AB, Elazar JM, and Majewski ML, Appl. Opt 37, 5271 (1998). 18286006 10.1364/ao.37.005271

[41] Chen AM, Xu HF, Jiang YF, Sui LZ, Ding DJ, Liu H, and Jin MX, Appl. Surf. Sci 257, 1678 (2010).

[42] Anisimov SI, Kapeliov BL, and Perelman TL, Sov. Phys. JETP 11, 945 (1967).

[43] Chekhovskoi VY, Tarasov VD, and Gusev YV, High Temp 38, 394 (2007).

[44] Lin Z, Zhigilei L, and Celli V, Phys. Rev. B 77, 75133 (2008).

[45] Assael MJ, Kalyva AE, Antoniadis KD, Michael Banish R, Egry I, Wu J, Kaschnitz E, and Wakeham WA,J. Phys. Chem. Ref. Data 39, 33105 (2010).

[46] Sarkar S, Jana PK, Chaudhuri BK, and Sakata H, Appl. Phys. Lett 89, 212905 (2006).

